# Architecture of the Short External Rotator Muscles of the Hip

**DOI:** 10.1186/s12891-019-2995-0

**Published:** 2019-12-20

**Authors:** Kevin C. Parvaresh, Charles Chang, Ankur Patel, Richard L. Lieber, Scott T. Ball, Samuel R. Ward

**Affiliations:** 1 Departments of Orthopaedic Surgery, University of California, 9500 Gilman Drive, La Jolla, San Diego, CA 92093-0863 USA; 2 Departments of Bioengineering, University of California, San Diego, USA; 3 Departments of Radiology, University of California, 9500 Gilman Drive, La Jolla, San Diego, CA 92093-0863 USA

**Keywords:** Hip, Muscle, Rotators, Architecture, Stability, Joint, Biomechanics, Fiber length

## Abstract

**Background:**

Muscle architecture, or the arrangement of sarcomeres and fibers within muscles, defines functional capacity. There are limited data that provide an understanding of hip short external rotator muscle architecture. The purpose of this study was thus to characterize the architecture of these small hip muscles.

**Methods:**

Eight muscles from 10 independent human cadaver hips were used in this study (*n* = 80 muscles). Architectural measurements were made on pectineus, piriformis, gemelli, obturators, quadratus femoris, and gluteus minimus. Muscle mass, fiber length, sarcomere length, and pennation angle were used to calculate the normalized muscle fiber length, which defines excursion, and physiological cross-sectional area (PCSA), which defines force-producing capacity.

**Results:**

Gluteus minimus had the largest PCSA (8.29 cm^2^) followed by obturator externus (4.54 cm^2^), whereas superior gemellus had the smallest PCSA (0.68 cm^2^). Fiber lengths clustered into long (pectineus - 10.38 cm and gluteus minimus - 10.30 cm), moderate (obturator internus - 8.77 cm and externus - 8.04 cm), or short (inferior gemellus - 5.64 and superior gemellus - 4.85). There were no significant differences among muscles in pennation angle which were all nearly zero. When the gemelli and obturators were considered as a single functional unit, their collective PCSA (10.00 cm^2^) exceeded that of gluteus minimus as a substantial force-producing group.

**Conclusions:**

The key findings are that these muscles have relatively small individual PCSAs, short fiber lengths, and low pennation angles. The large collective PCSA and short fiber lengths of the gemelli and obturators suggest that they primarily play a stabilizing role rather than a joint rotating role.

## Background

**Fig. 1:**
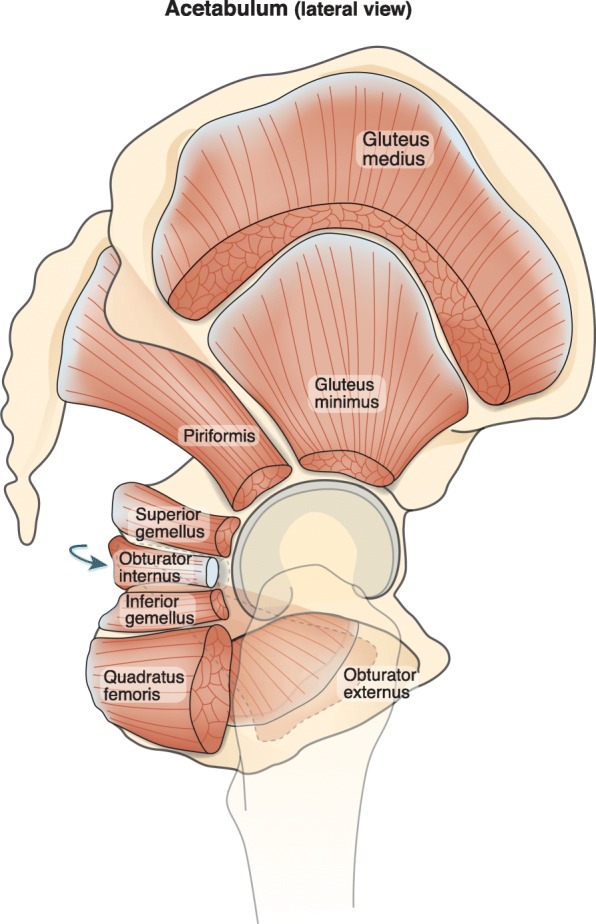
Lateral view of the hip with the selected muscles of the hip and gluteus medius illustrated

There is growing interest in hip joint function and pathology that has been accompanied by recent technological progress in biomechanical research. Much of the literature has focused on bony [1–3], labral [4–6], and capsular [7–9] morphology. Prior studies have shown that specific movement patterns are related to each of these pathologies [10–13]. Detailed architectural properties of the muscles surrounding the hip and the neighboring bony structures (Fig. 1) are essential to understanding the functional biomechanics of hip movement and stability.

Skeletal muscle architecture is defined as the arrangement of muscle fibers relative to the axis of force generation [14, 15]. Understanding muscle architecture is particularly important as it provides the best anatomical insight to predict muscle function [15]. To our knowledge, there is only one prior study that has evaluated the muscle architecture for the small rotational muscles of the hip [16]. In this study, Friedrich and Brand reported measurements for fiber length and physiological cross-sectional area (PCSA) for selected small rotational muscles of the hip. However, the study was extremely limited as it involved only two cadaveric specimens and did not normalize measurements based on sarcomere length. Sarcomere length measurement is critical because it allows normalized fiber length (Lf) and PCSA to be calculated. Without sarcomere length measurements, muscle measurements are distorted based on the fixation position of the cadaver. We previously showed that sarcomere length measurements eliminate this problem. These metrics are the only ones that are proportional to muscle excursion [17] and force generating capacity [18], respectively. Normalized architectural measurements are therefore required to understanding the role these muscles play in coordinating hip motion.

The purpose of this study was to measure the architectural properties of selected small muscles of the hip including the gluteus minimus, pectineus, piriformis, gemelli, obturators, and quadratus femoris. Our goal was to define the architectural properties of these muscles in order to better understand their functional role in hip joint biomechanics. We hypothesized that the architecture of these muscles would support their putative role in controlling joint position and providing stability.

## Methods

Whole cadaveric lower extremity specimens were obtained from the University of California, San Diego’s body donations program and were bisected along the midline. The regions of the hip and thigh were dissected through the deep fascia, and each muscle was visualized and obtained by removal from its most proximal origin to distal tendon attachment. Eight muscles (Table 1) from each of 10 formaldehyde-fixed human lower extremities (mean age ± standard deviation; 83 ± 9 years; male:female ratio, 5:5; height, 168.4 ± 9.3 cm; mass, 82.7 ± 15.3 kg, femoral head diameter 49.43 ± 1.1 mm) were carefully excised and stored in 1X phosphate-buffered saline (PBS).

**Table 1:** Muscle Architectural Properties

Muscle	Mass (g)	Muscle Length (cm)	Fiber Length (cm)	Ls (μm)	Pennation (deg)	PCSA (cm2)	LFN/LMN ratio
Pectineus ^a^	24.95 ± 2.31^c-f,h^	12.27 ± 0.52^c,e-g^	10.38 ± 0.53^b-g^	2.72 ± 0.05^b,d,e,g^	0.17 ± 0.80	2.27 ± 0.17^c-h^	0.85 ± 0.03^b,d,f^
Piriformis ^b^	19.10 ± 1.59^a,c-f,h^	11.83 ± 0.66^c,e-g^	8.37 ± 0.51^a,c,e,g,h^	2.47 ± 0.03^a,c,f,h^	2.83 ± 1.00	2.17 ± 0.15^c-h^	0.65 ± 0.03^a,c,e-h^
Superior Gemellus ^c^	3.52 ± 0.61^a,b,d,f-h^	5.83 ± 0.26^a,b,d,f-g^	4.85 ± 0.32^a,b,d,f-h^	2.85 ± 0.09^b,d-g^	0.00 ± 0.00	0.68 ± 0.12^a-b,d,f-h^	0.87 ± 0.02^b,d,f^
Obturator Internus ^d^	34.50 ± 2.60^a-c,e,h^	13.12 ± 0.27^c,e-g^	8.77 ± 0.34^a,c,g,h^	2.57 ± 0.03^a,c,h^	2.50 ± 1.12	3.76 ± 0.28^a-c,e-f,h^	0.63 ± 0.02^a,c,e-h^
Inferior Gemellus ^e^	5.90 ± 0.59^a,b,d,f-h^	6.37 ± 0.31^a,b,d,f,h^	5.64 ± 0.25^a,b,d,f,h^	2.45 ± 0.03^a,c,f,h^	0.50 ± 0.50	1.02 ± 0.11^a-b,d,f-g^	0.81 ± 0.03^b,d^
Obturator Externus ^f^	38.80 ± 2.50^a-c,e-h^	10.26 ± 0.51^a-h^	8.04 ± 0.42^a,c,e,g-h^	2.64 ± 0.04^b,c,e^	1.67 ± 1.24	4.54 ± 0.16^a-e,g-h^	0.77 ± 0.03^a-d^
Quadratus Femoris ^g^	25.29 ± 2.89^c,e,f,h^	7.20 ± 0.41^a-d,f,h^	6.37 ± 0.34^a-d,f,h^	2.53 ± 0.02^a,c,h^	0.00 ± 0.00	3.75 ± 0.42^a-c,e-f,h^	0.83 ± 0.02^b,d^
Gluteus Minimus ^h^	92.15 ± 10.87^a-g^	13.11 ± 0.85^c,e-g^	10.30 ± 0.77^b-g^	2.79 ± 0.06^b,d,e,g^	1.67 ± 1.45	8.29 ± 0.51^a-g^	0.81 ± 0.01^b,d^

Values are expressed as mean ± standard error. *L*_*s*_ Sarcomere Length. *PCSA* Physiologic Cross Sectional Area. *L*_*m*_ Muscle length normalized to neutral sarcomere length of 2.7 μm

^s^ Significantly different than pectineus, ^s^ Significantly different than piriformis, ^s^ Significantly different than superior gemellus, ^d^ Significantly different than obturator internus, ^e^ Significantly different than inferior gemellus, ^f^ Significantly different than obturator externus, ^g^ Significantly different than quadratus femoris, ^h^ Significantly different than gluteus minimus

Muscle architectural measurements were made based on the same methods described by Sacks and Roy [19], modified by Lieber et al. [20], and adapted for the lower extremity by Ward et al. [21]. Briefly, muscle length (Lm) was measured as the distance from most proximal fibers to the most distal fibers. Raw fiber length (Lf′) was measured for each muscle in 3 regions; proximal, middle, and distal; using a digital caliper (accuracy, 0.01 mm). Surface pennation angle was measured with a goniometer as the angle between the fibers and the distal tendon. Values for normalized fiber length (Lf) were calculated based on the following equation [22]:

$$ \mathrm{Lf}=\mathrm{L}{\mathrm{f}}^{\prime}\left(2.7\ \upmu \mathrm{m}/\mathrm{Ls}\right) $$

where Ls is the measured sarcomere length and 2.7 μm is the optimum sarcomere length for human muscle [22]. Normalizing fiber length is key as it allows for comparisons among muscles fixed in various degrees of tension and sarcomere lengths [23]. Normalized Lm was calculated using a similar equation. The Lf/Lm ratio was also determined to assess excursion design comparisons across muscles [15]. PCSA was calculated according to the following equation: [18].

$$ \mathrm{PCSA}\ \left({\mathrm{cm}}^2\right)=\left(\mathrm{M}\ \left(\mathrm{g}\right)\times \cos \uptheta \right)/\left(\uprho\ \left(\mathrm{g}/{\mathrm{cm}}^3\right)\times \mathrm{Lf}\ \left(\mathrm{cm}\right)\right) $$

where M is mass, θ is pennation angle, and ρ is muscle density (1.056 g/cm3) [24], accounting for dehydration that occurs during fixation.

Multiple measurements were made on each muscle (n= > 3), then averaged for each sample, yielding grand means which are presented. All data are reported as mean ± standard error unless otherwise noted. Between-muscle and between-muscle group comparisons of mass, mean fiber length, and total PCSA were made with one-way ANOVAs after confirming the assumptions of normality and homogeneity of variances were met. Comparisons to gluteus medius and maximus were made using previously reported data from similarly aged specimens [21]. Post hoc Tukey’s tests were used to identify specific muscle differences. All analyses were performed using SPSS® software (Version 20.0; SPSS Inc., Chicago, IL). Significance was set to *p* < 0.05 for the ANOVA and post hoc tests.

## Results

**Fig. 2:**
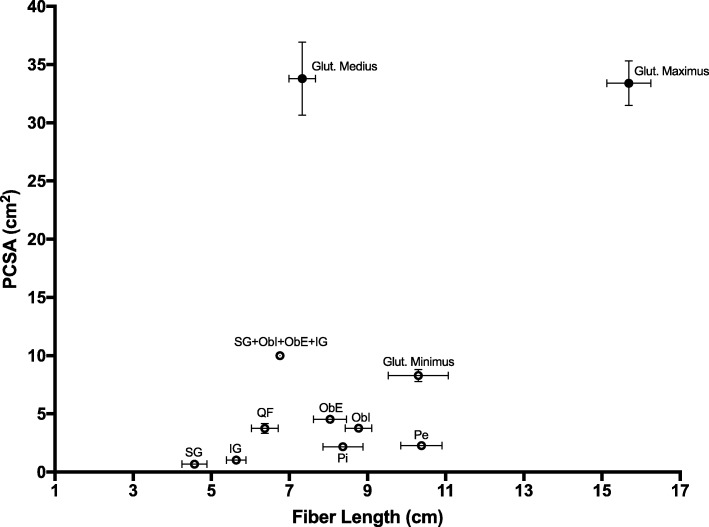
Scatterplot of fiber length versus PCSA for the selected muscles reported here (open circles) in comparison to the gluteus medius and maximus data collected previously (filled circles) [21]. The combined value for the short external rotators (gemelli and obturators) is displayed as well. QF = quadratus femoris, ObE = obturator externus, ObI = obturator internus, SG = superior gemellus, IG = inferior gemellus, Pi = piriformis, Pe = pectineus

Architecturally, there were many significant differences among the selected muscles in terms of mass, Lm, Lf, Ls, PCSA and Lf/Lm ratio (*p* < 0.001, Table 1). Importantly, values for PCSA clustered into very small (superior and inferior gemelli), small (pectineus and piriformis), moderate (obturator internus and externus and quadratus femoris) or large (gluteus minimus) values. However, the large gluteus minimus PCSA (8.29 ± 0.51 cm^2^) was still much smaller than the value previously reported for gluteus medius (33.8 ± 14.4 cm^2^) and gluteus maximus (33.4 ± 8.8 cm^2^) [21] (Fig. 2). The collective PCSA of the gemelli and obturators (10.0 ± 0.67 cm^2^) was larger than the gluteus minimus.

Values for fiber length also clustered into very short (superior gemellus, inferior gemellus, and quadratus femoris), short (piriformis, obturator internus and externus) or moderate (pectineus and gluteus minimus) lengths. On average, these values were similar to the short fiber length observed for gluteus medius (7.33 ± 1.57 cm) and shorter than the long fiber length observed for gluteus maximus (15.69 ± 2.57 cm) [21] (Fig. 2). Average Ls (2.63 μm) tended to be shorter than optimal length (2.7 μm), which was not surprising given the externally rotated position of the hips at the time of fixation. Fiber length to muscle lengths ratios tended to be long with the exception of piriformis (0.65 ± 0.03) and obturator internus (0.63 ± 0.02). There were no significant differences in pennation angles which were nearly zero for all muscles.

## Discussion

In this study we report the detailed the architectural properties of the rotational muscles of the hip, including pectineus, piriformis, gemelli, obturator internus and externus, quadratus femoris, and gluteus minimus, corrected for muscle sarcomere length. The key findings were the relatively small individual PCSAs, short fiber lengths, short sarcomere lengths, and uniformly low pennation angles. To our knowledge there are no prior publications on the properties of these selected hip muscles utilizing sarcomere length to provide normalized values for fiber length and PCSA.

In a comprehensive literature review of the short external rotators of the hip, Yoo et al. [25] found only one published article containing quantitative values of hip muscle architecture [16]. In that study, Friederich and Brand reported architectural measurements from two cadaveric specimens without sarcomere normalization. Regarding the short external rotators, they found the PCSAs of the piriformis (20.54 cm^2^) and quadratus femoris (21.00 cm^2^) were the largest, by approximately a 4 fold greater magnitude. Our data showed a similar range of distribution of PCSAs between the short external rotator muscles, but our PCSA values were significantly smaller (Fig. 2). The lack of sarcomere length measurements from their study makes it difficult to reconcile our data, highlighting the importance of this normalizing measurement for comparing muscle architecture because most sarcomere lengths they measured were below optimum.

This concept may be supported by comparing our data to the normalized data of Wickiewicz et al. [26]. In their pilot study, they report normalizing muscle architecture data to sarcomere length. Although their sarcomere length was based on an average from a separate study and not a direct measurement, it still provides a baseline for comparison. Their average measurements for pectineus muscle length (12.30 cm), fiber length (10.43 cm), pennation angle (0 degrees), and PCSA (2.9) are nearly identical to our values (Table 1). Although pectineus was the only muscle available for direct comparison, these findings underscore the importance of normalizing data in comparing muscle architecture.

Our data provide a number of insights into the design of the hip short external rotators. All muscles exhibited an almost parallel pennation angle of 0 degrees, suggesting force generation is maximized to act in a single axis of rotation. This may be helpful in maintaining low muscle mass and PCSA in constrained regions of the hip while still allowing sufficient force generation. From gross dissection, we know that the superior gemellus, obturator internus, inferior gemellus, and obturator externus are essentially fused. If these muscles are considered as a single functional unit, their collective PCSA becomes functionally relevant (Fig. 2). In fact, their combined PCSA exceeds that of gluteus minimus. With the addition of quadratus femoris and piriformis, the collective “short external rotators” become a substantial force-producing unit. Considering them as a unit with a large PCSA and short fiber lengths, their design features correspond to a stabilizing role [27].

Although we did not directly measure joint geometry, these architectural data may be combined with known values previously reported to evaluate their role in muscle-joint kinematics. Due to the short external rotators’ close proximity to the axis of rotation, muscle length does not change substantially relative to joint position and moment arms remain oriented toward external rotation [28]. Unlike the gluteal muscles, the short external rotators may therefore rotate the hip relatively independent of sagittal and coronal motion. Such independent movement provides valuable rotational control without otherwise affecting joint position. When combined with their rotational antagonists (gluteus minimus, pectineus, and adductors), these muscles appear to provide a stabilizing role to the hip joint [29]. With simultaneous internal and external rotational contraction, a medial compressive force is created to balance the lateralizing force of the abductors. Such balance may facilitate dynamic stabilization of the hip joint, though further studies are necessary to validate these hypotheses.

Additionally, our findings have implications for current surgical approaches to the hip. Decisions to release the short external rotators during hip surgery should represent a balance between achieving adequate surgical exposure and preserving soft tissue anatomy, which may lead to less post-operative pain, faster rehabilitation, and a more stable joint [30]. During traditional posterior approaches to the hip, the short external rotators are often sacrificed. Early in the practice of total hip arthroplasties, leaving the short external rotators unrepaired was believed to have no adverse effect on hip stability [8, 31]. The importance of these structures has become apparent in the recent years, however, as other reports have shown that adequate repair of the posterior structures greatly decreases the future risk of hip instability caused by soft-tissue attenuation [32, 33]. While recent meta-analyses have shown that surgical approach does not affect dislocation rate [34], few studies directly measured muscle function following surgery. Evolving techniques including the direct superior approach, which spares the external rotators, may offer a functional advantage [35], but long-term comparison studies are lacking. Further research should be dedicated to assessment of hip muscle function following hip surgery.

This study has several limitations. Fixation position was in an externally rotated joint configuration and may not reflect the clinically accepted definition of a neutral hip joint angle which is 0° abduction, flexion, and rotation. However, normalization of results with sarcomere length removes variation associated with position and therefore positioning should not significantly affect the results. Second, the advanced age of the cadaveric specimens may have led to lower PCSAs than would otherwise be observed in younger patients, but still likely provide a baseline for functional predictions and are comparable among muscles. Future studies may expand on these data for functional evaluation such as electromyographic studies of activation patterns during various movements and activities.

## Conclusions

In summary, these findings characterize the architecture of selected muscles of the hip. These data support the hypothesis that these muscles act as dynamic stabilizers. Moreover, they highlight the functional importance of these muscles relative to hip pathology, surgery, and rehabilitation. We suggest that these data be expanded in the future to characterize the dynamic interactions among these muscles and other extra- and intra-articular structures as well as muscle adaptations to immobilization and pathology to further our knowledge of hip biomechanics.

## Data Availability

The datasets analyzed during the current study are available as a supporting file or from the corresponding author on reasonable request.

## Abbreviations

ANOVA: Analysis of variance
Lf: Normalized fiber length
Lf’: Raw fiber length
Lm: Muscle length
Ls: Sarcomere length
M: Mass
PBS: Phosphate-buffered saline
PCSA: Physiological cross-sectional area
θ: Pennation angle
ρ: Muscle density
